# From commitments to action: the first global cervical cancer elimination forum

**DOI:** 10.1016/j.lana.2024.100812

**Published:** 2024-06-12

**Authors:** Maribel Almonte, Andre Michael Ilbawi, Mauricio Maza, Marion Saville, Raul Murillo, Mathilde Forestier, Paul Bloem, Silvana Luciani, Bente Mikkelsen

**Affiliations:** a Department of Non-communicable Diseases, Rehabilitation and Disability, World Health Organization, Geneva, Switzerland; b Department of Non-communicable Diseases, Pan American Health Organization, Washington, DC, USA; c The Australian Centre for Prevention of Cervical Cancer, Melbourne, VIC, Australia; d Pontificia Universidad Javeriana, Bogotá, Colombia; e Department of Immunization, Vaccines & Biologicals, World Health Organization, Geneva, Switzerland

On 5–7 March, 2024, the first Global Cervical Cancer Elimination Forum brought together leaders from Ministries of Health, development partners and multilateral institutions, United Nations (UN) agencies and civil society to catalyse national and global momentum to eliminate this preventable disease, capitalizing on available technologies of human papillomavirus (HPV) vaccines, HPV tests and ablative treatment devices. The partners pledged nearly US$600 million to eliminate cervical cancer.^1^ This was a game-changing commitment which gives hope for women all over the world, particularly in African countries.

Cervical cancer is the fourth most commonly occurring cancer globally and the fourth leading cause of cancer death among women, accounting for around 662 000 new cases and around 349 000 deaths globally in 2022. It is the most commonly diagnosed cancer in women in 25 countries, many of which are in sub-Saharan Africa.^2^

The Director General of the World Health Organization (WHO) launched the Cervical Cancer Elimination Initiative in 2018, and in 2020, WHO Member States adopted the WHO Global Strategy of Cervical Cancer Elminitation.^3^ The threshold to reach elimination is four invasive cervical cancers per 100 000 women yearly, and to remain on the path toward elimination, countries must meet these targets by 2030:

• 90% of girls fully vaccinated against HPV by age 15;
• 70% of women screened using a high-performance test by ages 35 and 45;
• 90% of women identified cervical disease receiving treatment and care.

Yet, in spite of strong political commitments, progress has been slow: while more than 50 additional countries introduced the vaccine since 2018, only 21% of girls worldwide were vaccinated with the first dose against HPV in 2022. Most countries are far from achieving 70% screening participation and treatment services remain widely inaccessible. Although the cost-effectiveness of these interventions is extensively documented, a primary challenge, has been the failure to commit and mobilize necessary fiscal resources, resulting in insufficient availability of HPV vaccines, HPV tests and precancer treatment devices, as well as in limited cancer management capacity including lack of trained workforce and of diagnostic and treatment services.

It is against this backdrop that the outputs from this Global Forum are extraordinary. Governments and partners made major policy, programmatic and financial commitments, including nearly US$600 million in funding, while showcasing the feasibility of implementing HPV vaccination, HPV screening and affordable treatment in low- and middle-income countries (LMIC).^1^ We are rightly pivoting toward more comprehensive and innovative approaches to financing cervical cancer elimination, relying on diverse and blended capital and better articulating investment opportunities for investors.

The Global Forum was hosted by Colombia, in the Latin America and Caribbean (LAC) region, where coverage for vaccination and screening remain below targets and elimination is not yet within reach in any country. However, LAC may be the region best situated to feature substantive progress because of early and wide adoption of emerging “game-changing” policies, including scale-up of one-dose HPV vaccination, HPV testing on self-collected samples and increased use of affordable ablative treatment devices.

In 2008, the first HPV vaccination programme in the LAC region started in Panama, targeting girls aged 10–11 years with a 3-dose vaccine schedule. Currently, 32 (97%) countries in the region have implemented HPV vaccination, and 11 of them have already adopted the single-dose HPV vaccine schedule recommended by WHO in 2022.^4^^,^^5^

In 2012, and with further evidence in 2021, WHO issued guidelines for screening and treatment of precancer lesions to prevent cervical cancer^6^ recommending to screen with HPV tests on self- or provider-collected samples. Fourteen LAC countries have started transition to HPV-based screening, 7 of them are either piloting or using HPV testing on self-collected samples regionally or nationally.^7^^,^^8^ However, despite increased use of ablative treatment devices, linkage to treatment remains limited, highlighting the need to reinforce that both screening uptake and adequate treatment are indispensable.

LAC countries are also investing in services to manage invasive cervical cancer. LAC is the region most likely to include cervical cancer in its national benefit packages and with the highest coverage.^9^ Commitment to implement new policies considering women’s and workforce perspectives and the role of professional and civil societies has been remarkable over the last decade. This has enabled, as an example, a successful recovery of HPV vaccination coverage after the COVID-19 pandemic (coverage increased from 53% in 2021 to 68% in 2022, Fig. 1).

**Fig. 1:**
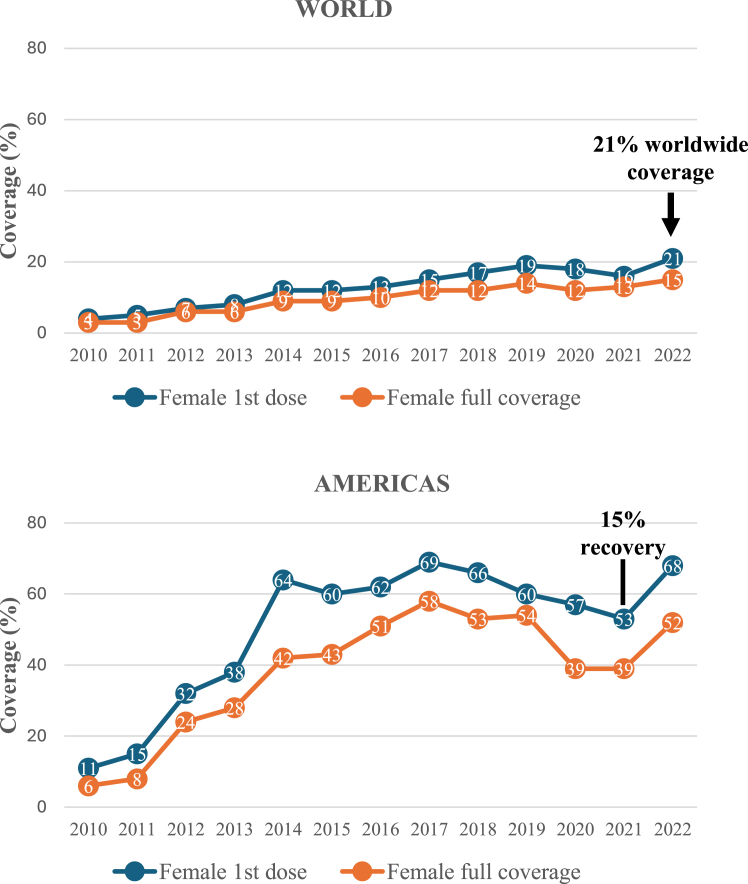
Early signs of recovery of human papillomavirus (HPV) vaccination coverage among 10–14 year-old girls post-COVID-19 in the World and the Region of the Americas (including North America). Observed coverage fluctuations are mainly driven by Latin America and the Caribbean (LAC) as estimates from North America have been stable during the 2021–2022 period.^5^

Progression to elimination targets is feasible in LAC and in every country around the world. With this Global Forum, we now have more political and technical commitment, more partners, more funding. As a follow-up, the partners are expected to recognize their commitments within their mandate and in alignment with the Global Strategy of Cervical Cancer Elimination. Together, we can be the generation that leads the movement of elimination so that none of our children or grandchildren ever experience the suffering of this preventable disease.

## Contributors

MA, AMI, and BM conceived the article; MA and AI wrote the article; MF and PB provide data and figures on HPV vaccination; MA, AMI, MM, MS, RM, MF, PB, SL, and BM reviewed the article.

## Declaration of interests

We declare no competing interests.
